# Modulation of Gut Microbiota by Red Ginseng Extract Powder and Dietary Fiber in Obese Mice: Identification of Key Microbial Candidates

**DOI:** 10.4014/jmb.2506.06016

**Published:** 2025-08-18

**Authors:** Jonghyeok Shin, Do Young Jin, Seung-Hwan Seo, Hye-Young Yu, Sang-Kyu Kim, Seung-Ho Lee, Eun-Hee Shin, Jun-Seob Kim

**Affiliations:** 1 Synthetic Biology Research Center and the K-Biofoundry, Korea Research Institute of Bioscience and Biotechnology (KRIBB), Daejeon 34141, Republic of Korea; 2 Department of Nano-Bioengineering, Incheon National University, Incheon 22012, Republic of Korea; 3 Department of Tropical Medicine and Parasitology, Seoul National University College of Medicine, Seoul 03080, Republic of Korea; 4 Institute of Endemic Diseases, Seoul National University Medical Research Center, Seoul 03080, Republic of Korea; 5 R&D Headquarters, Korea Ginseng Corporation, Gwacheon 13840, Republic of Korea; 6 Seoul National University Bundang Hospital, Seongnam 13620, Republic of Korea; 7 Research Institute for New Drug Development, Incheon National University, Incheon 22012, Republic of Korea

**Keywords:** Red ginseng, gut microbiota, obesity, prebiotics, metabolic health

## Abstract

Red ginseng extract powder (RGEP) and red ginseng dietary fiber (RGDF) contain bioactive components with potential prebiotic effects. As gut microbiota plays a critical role in obesity and is influenced by prebiotics, we investigated the effects of RGEP and RGDF supplementation on gut microbiota diversity, composition, and metabolic functions in diet-induced obese mice. RGEP and RGDF supplementation altered gut microbiota composition, increasing beneficial bacteria such as *Lactobacillus*, *Roseburia*, and *Akkermansia*. Alpha diversity analysis showed an increase in microbial richness, particularly in the high-dose RGDF group, whereas beta diversity analysis confirmed a distinct separation between red ginseng-fed groups and obesity models. Functional pathway analysis revealed that supplementation with RGEP and RGDF enhanced short-chain fatty acid (SCFA) metabolism, lipid metabolism, and anti-inflammatory metabolism, suggesting modulation of gut microbial functional profiles. These findings suggest that RGEP and RGDF contribute to gut microbiota modulation by enhancing microbial diversity, promoting SCFA metabolism, and suppressing pro-inflammatory bacterial taxa. While only gut microbiota profiles were analyzed, the observed restoration of microbial balance suggests a potential contribution of red ginseng components to host metabolic health, which warrants further investigation. Further studies are needed to validate these findings in human trials and elucidate the underlying molecular mechanisms.

## Introduction

Obesity is a major global health concern, contributing to metabolic disorders such as insulin resistance, type 2 diabetes, and cardiovascular diseases [1, 2]. Recent studies suggest that gut microbiota plays a crucial role in obesity and its associated metabolic dysfunctions [3-5]. In obesity, gut microbiota dysbiosis occurs, characterized by a decrease in microbial diversity and an imbalance between beneficial and harmful bacterial populations. These microbial alterations contribute to fat accumulation, increased inflammation, and impaired energy homeostasis, thereby exacerbating metabolic disorders associated with obesity [6, 7]. As a result, modulating the gut microbiota to restore balance has emerged as a promising strategy for treating obesity and improving metabolic health.

Red ginseng (*Panax ginseng* Meyer) is a processed form of ginseng that undergoes steaming and drying, enhancing its bioactive properties. Traditionally, red ginseng has been recognized for its various health benefits, including immune modulation, antioxidant activity, anti-inflammatory effects, and blood glucose regulation [8]. In particular, recent studies have also highlighted the potential role of red ginseng in obesity and diabetes management, improving metabolic health by regulating glucose and insulin levels [9, 10]. These metabolic effects are thought to be linked to the modulation of gene expression involved in energy metabolism [11]. Furthermore, red ginseng has been reported to impact gut microbiota composition, with alterations observed in the gut microbial communities of individuals with obesity [12, 13].

Conventionally, red ginseng is consumed as a water extract, which contains a high concentration of ginsenosides. However, during the extraction process, a significant portion of the plant material is discarded as residue, despite retaining bioactive compounds such as unextracted ginsenosides, acidic polysaccharides, essential minerals, and dietary fiber. To utilize these underused components, red ginseng residues have been explored for their potential applications in pharmaceuticals, functional foods, and cosmetics [14]. Building on this approach, red ginseng extract powder (RGEP) and red ginseng dietary fiber (RGDF) are novel formulations designed to maximize the functional potential of red ginseng by incorporating both its water-soluble and fiber-rich components [15, 16]. RGDF, derived from fiber-rich ginseng residues, has recently been recognized for its ability to modulate gut microbiota and potentially function as a prebiotic by selectively supporting beneficial bacterial populations [15, 17, 18]. RGEP, a concentrated ginseng extract, is also expected to exhibit similar microbiome-modulating effects due to its high content of bioactive compounds, which may promote beneficial microbial growth while exerting anti-inflammatory and metabolic benefits [16]. Unlike traditional red ginseng extracts, RGEP and RGDF offer an enhanced functional spectrum by utilizing both ginsenoside-rich extracts and fiber-based prebiotic components, ensuring a dual-action approach for regulating metabolism and gut microbiota.

Previous studies have demonstrated that RGEP and RGDF contribute to maintaining intestinal immune homeostasis and improving gut barrier function in diet-induced obese (DIO) mice. RGEP and RGDF were shown to reduce inflammatory cytokines, enhance mucosal barrier integrity, and restore gut functional homeostasis [16]. However, the direct impact of these red ginseng-derived components on gut microbiota composition, microbial diversity, and functional metabolic pathways remains unclear. Given the interplay between gut inflammation and microbiome composition, it is crucial to determine how RGEP and RGDF affect microbial communities and their metabolic functions, which could further explain their beneficial role in obesity management.

This study aimed to investigate the effects of RGEP and RGDF on gut microbiota composition in DIO mice. Specifically, it examined whether RGEP and RGDF influence microbial diversity and composition while identifying key microbial candidates contributing to metabolic improvements. Additionally, the study evaluates the impact of RGEP and RGDF on SCFA production and β-glucuronidase activity, which serve as important indicators of gut metabolic health. Furthermore, it explored the potential of RGEP and RGDF as prebiotic agents capable of restoring gut homeostasis and alleviating obesity-associated dysbiosis.

## Materials and Methods

### Dosage Determination and Administration of RGEP, RGDF, and Fructooligosaccharide in Mice

RGEP, RGDF, and fructooligosccharide (FOS) were provided by Korea Ginseng Corporation (KGC). The dosages of RGEP and RGDF were selected based on human daily intake levels of 2 g/day (for RGEP) and 4 g/day (for RGDF), which are standard for commercially available products. These were scaled to murine equivalents (205–1,620 mg/kg) using standard allometric conversion factors for a 60 kg adult, as described previously. These doses were converted to mouse-equivalent doses using allometric scaling, resulting in: RGEP: 205 mg/kg (L), 410 mg/kg (M), 820 mg/kg (H), RGDF: 410 mg/kg (L), 820 mg/kg (M), 1,640 mg/kg (H), FOS: 820 mg/kg [16]. The final doses were prepared weekly by dissolving RGEP, RGDF, and FOS in distilled water (DW) according to the average body weight of each group. All animal procedures were reviewed and approved by the Institutional Animal Care and Use Committee (IACUC) of Seoul National University Hospital under protocol number 23-0037-S1A0. The experiments were carried out in compliance with internationally accepted ethical standards, including those of the Association for Assessment and Accreditation of Laboratory Animal Care International (AAALAC International). Animals were housed and treated in accordance with the Animal Protection Act and the Laboratory Animal Act of the Republic of Korea.

### Experimental Animals and Grouping

This study followed a previously established protocol for animal grouping and diet administration [16]. 7-wk-old male C57BL/6 mice were obtained from Orient Bio, Inc., (Republic of Korea) and housed in a biosafety level-2 facility at the Seoul National University Hospital Biomedical Research Institute. After a 1-week acclimation period, mice were fed either a high-fat diet (HFD, 60 kcal% fat, D12492) or a low-fat diet (LFD, 10 kcal% fat, D12450B) for 6 weeks. Mice with a body weight exceeding 40 g were classified as the obesity group, with an average weight of 41.78 g for the 4-week intake group and 41.94 g for the 8-week intake group. The normal control group fed LFD had an average body weight of 27.04 g (4-week group) and 27.58 g (8-week group). The experimental groups were divided as follows: LFD (normal control), HFD (positive control), HFD + FOS (820 mg/kg), HFD + RGEP (205, 410, or 820 mg/kg), and HFD + RGDF (410, 820, or 1,640 mg/kg). For both RGEP and RGDF, the three concentrations were designated as low (L), medium (M), and high (H), respectively, and are referred to throughout the manuscript as RGEPL, RGEPM, RGEPH and RGDFL, RGDFM, RGDFH. Mice were orally administered 300 μl of RGEP, RGDF, or FOS once daily for either 4 or 8 weeks, following a regimen previously established in prior studies. At the end of the experiment, all mice were anesthetized, and biological samples were collected, including blood (via cardiac puncture), feces, urine, intestinal tissues (from the stomach to anus), and mesenteric lymph nodes (MLNs). These samples were analyzed using a standardized assays related to intestinal health to evaluate the effects of RGEP and RGDF supplementation. While multiple biological samples including blood, urine, and intestinal tissues were collected during the experiment, the present study focuses solely on fecal microbiota analysis. Detailed analyses of host systemic and immunological parameters using the other sample types are available in a separate report [16].

### DNA Extraction and Sequencing

Total genomic DNA was extracted from each stool sample using the QIAamp PowerFecal Pro DNA Kit (QIAGEN, Cat. No. 51804, Germany) according to the manufacturer’s recommendations. To disrupt microbial cells in the stool samples, each stool sample was combined with 800 μl of buffer in a PowerBead Pro tube. The suspensions were vortexed horizontally at maximum speed for 20 min until homogenized. After centrifugation at 15,000 ×*g* for 1 min, the supernatants were collected and mixed with 200 μl of CD2 buffer to remove inhibitors. The lysates were centrifuged again at 15,000 ×*g* for 1 min, and the supernatants were collected. These supernatants were then mixed with 600 μl of CD3 buffer to adjust the salt concentration of the DNA solution, allowing for more specific DNA binding to the column. The lysates were applied to MB spin columns and centrifuged at 15,000 ×*g* for 1 min. The columns were washed twice with EA and C5 wash buffer, respectively. Finally, DNA was eluted by adding 70 μl of C6 solution and centrifuging at 15,000 ×*g* for 1 min. The hypervariable V4 region of the microbial 16S rRNA gene was amplified by PCR using the following primers: forward primer 515F (5'-GTGCCAGCMGCCGCGGTAA-3') and reverse primer 806R (5'‐GGACTACHVGGGTWTCTAAT‐3'). Sequencing was performed using the Illumina MiSeq platform (Illumina Inc., USA) with 250 bp paired-end reads.

### Data Processing

Demultiplexed FASTQ datasets were analyzed using QIIME 2 version 2024.10 [19]. Raw sequences were imported into the framework using the PairedEndFastqManifestPhred33V2 format. Primer sequences from both forward and reverse reads were trimmed using the q2-cutadapt plugin [20]. Denoising was performed using the q2-dada2 plugin, which filters out singletons, chimeras, and sequencing errors to generate amplicon sequence variants (ASVs) [21]. Samples were rarefied to a maximum depth of 67,485 sequences using the alpha rarefaction tool. The microbial diversity analyses, including alpha (*e.g.*, Shannon diversity) and beta diversity metrics (*e.g.*, Bray-Curtis dissimilarity), were performed using the core-metrics-phylogenetic pipeline in QIIME 2. Taxonomy classification of ASVs was conducted against the SILVA 138.2 database [22]. Differentially abundant features across sample groups were identified using Analysis of Compositions of Microbiomes with Bias Correction (ANCOM-BC) [23].

### Functional Pathway Analysis

Functional pathway prediction was conducted using PICRUSt2 version 2.5.3 [24]. ASV representative sequences and a feature table containing operational taxonomic units (OTU) and taxonomy data were phylogenetically placed into a reference tree using place_seqs.py. The sequence placement outputs were then used to perform hidden state prediction via hsp.py, which inferred the functional potential of the metagenome by predicting gene family abundances (*e.g.*, Enzyme Commission numbers and KEGG Orthologs). Finally, the pathway-level abundances were predicted using pathway_pipeline.py, followed by the annotation with descriptions from the MetaCyc database.

### Statistical Analysis

Alpha diversity indices were compared among groups using one-way Analysis of Variance (ANOVA) in R version 4.4.3 with the multcomp R package [25]. Statistical significance for beta diversity comparisons was assessed using Permutational Multivariate Analysis of Variance (PERMANOVA), in the q2-diversity plugin of QIIME 2. Visualization of results included relative abundance bar plots, volcano plots for differential features, box plots for alpha diversity metrics, and Principal Coordinates Analysis (PCoA) plots for beta diversity distances, all created using the ggplot2 R package [26]. Heatmaps of predicted functional pathways from PICRUSt2 results were generated using the pheatmap R package.

## Results

### Animal Grouping and Diet Administration

**Fig. 1:**
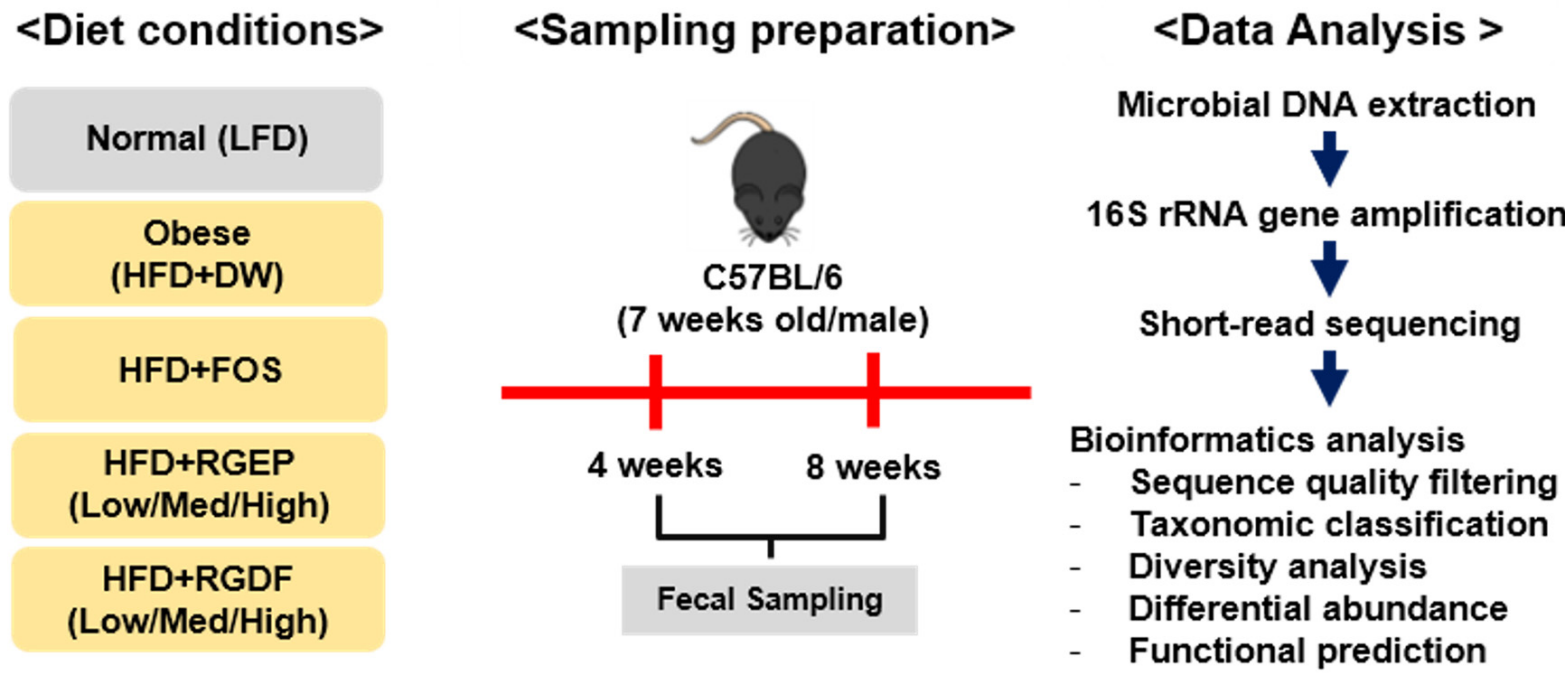
Schematic overview of the experimental design. Obesity was induced in mice using a high-fat diet (HFD), followed by oral administration of red ginseng extract powder (RGEP) or red ginseng dietary fiber (RGDF) at three concentrations—Low (L), Medium (M), and High (H)—in combination with the HFD. Fructooligosaccharide (FOS) was included as a positive control due to its known anti-obesity effects. Mice fed a low-fat diet (LFD) served as a normal control. Fecal samples were collected at weeks 4 and 8 to evaluate the gut microbiota composition. Microbial DNA was extracted and analyzed following a standardized pipeline, including 16S rRNA gene sequencing and bioinformatic processing. Abbreviations: LFD, low-fat diet; HFD, high-fat diet; RGEP, red ginseng extract powder; RGDF, red ginseng dietary fiber; FOS, fructooligosaccharide; L, low dose; M, medium dose; H, high dose.

The effects of RGEP and RGDF diets on gut microbiota composition were investigated in mice. C57BL/6 male mice (7 weeks old) were subjected to an HFD and administered various dietary treatments over 8 weeks. This manuscript focuses specifically on fecal microbiota analysis from these animals. This study reports findings based on fecal microbiota analysis. Other biological samples were also collected during the experiment, and relevant host-level analyses have been published separately [16]. Fecal samples were collected at weeks 4 and 8 to analyze and compare gut microbiota composition, using standard DNA extraction and 16S rRNA gene sequencing protocols. FOS was selected as a positive control based on its well-documented prebiotic activity. It is selectively fermented by beneficial microbes such as *Lactobacillus* and *Bifidobacterium* sp., which are known to enhance host metabolic health [27-29] (Fig. 1).

### Validation of Sequencing Depth through Rarefaction Curve Analysis

To ensure the reliability of the sequencing data for evaluating microbial diversity, rarefaction curve analysis was performed. Rarefaction curves represent the relationship between sequencing depth (*i.e.*, number of reads) and observed species richness, visually indicating whether additional sequencing would yield new taxa [30]. A total of 11,061,737 high-quality 16S rRNA gene sequences were obtained across 126 fecal samples, with individual samples yielding between 32,970 to 85,537 reads. The sequences were clustered into 1,888 OTUs with each sample containing 97 to 250 OTUs. Rarefaction curves for all groups initially showed a steep increase and gradually plateaued, indicating that the sequencing depth was adequate to capture the majority of bacterial diversity in each sample (Fig. S1). No substantial differences in curve saturation were observed among the groups, suggesting uniform sequencing performance. These results validate the robustness of the sequencing data and support its suitability for subsequent analyses of alpha and beta diversity.

### Alteration of Gut Microbiota Composition Following Administration of RGEP and RGDF

To investigate the overall composition of the gut microbiota, we analyzed the relative abundance of bacterial taxa at both the phylum and family levels across experimental groups. At the phylum level, *Bacillota* and *Bacteroidota* were consistently identified as the predominant phyla across all groups and time points (Fig. S2). At the family level, *Lachnospiraceae*, *Bacteroidaceae*, *Streptococcaceae*, and *Lactobacillaceae* were the dominant families observed in all groups.

**Fig. 2:**
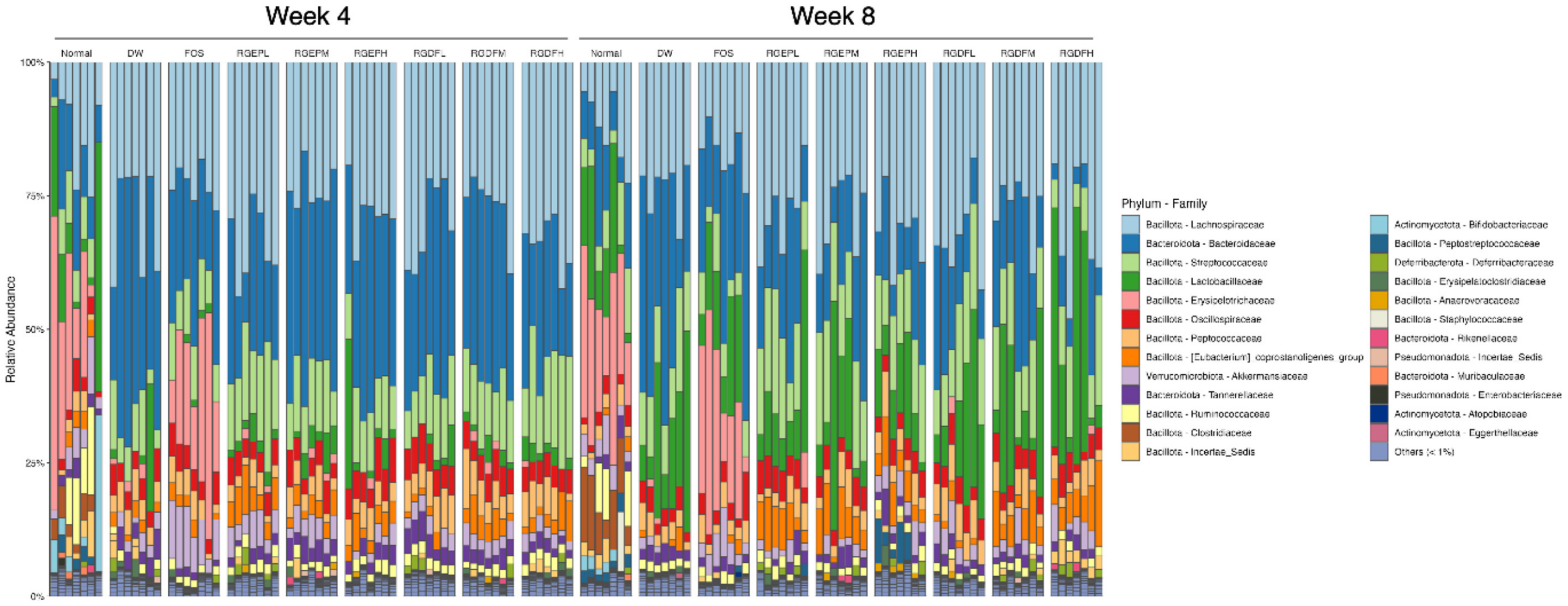
Relative abundance of gut microbiota at the family level in each experimental group. Stacked bar plots represent the microbial composition of individual mouse fecal samples at week 4 (left half) and week 8 (right half). For each timepoint, groups are ordered as follows (left to right): Normal, DW, FOS, RGEPL, RGEPM, RGEPH, RGDFL, RGDFM, and RGDFH. Each bar corresponds to a single mouse, and colors represent bacterial families. Due to the high number of taxa and individuals, the plot is intended to convey broad compositional trends rather than individual taxa differences.

Notably, the relative abundance of *Erysipelotrichaceae*, a bacterial family associated with hepatic cholesterol metabolism, was higher in mice fed a normal diet without red ginseng and in those receiving HFD supplemented with FOS, compared to other groups (Fig. 2).

In contrast, the *Eubacterium coprostanoligenes* group, known for converting cholesterol into non-absorbable coprostanol, was significantly enriched in red ginseng-treated groups (Fig. S3A and S3B) [31]. These results suggest that RGEP and RGDF supplementation may act as prebiotics, selectively modulating gut microbiota by promoting cholesterol-metabolizing bacteria such as *Eubacterium coprostanoligenes* while avoiding the *Erysipelotrichaceae*-associated dysbiosis observed in FOS-supplemented groups.

### Differential Abundance Barplot of DIO Mice Fed with RGEP or RGDF

To identify specific bacterial taxa that were significantly modulated by red ginseng supplementation, we performed ANCOM-BC. This statistical method accounts for the compositional nature of microbiome data and yields robust, FDR-adjusted q-values. Differential abundance analysis was conducted by comparing the obesity control group (HFD + DW) with RGEP- and RGDF-treated groups (low, medium, and high doses) at both weeks 4 and 8 time points.

**Fig. 3:**
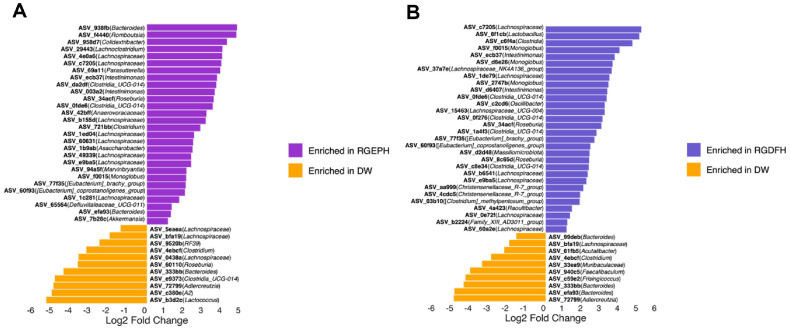
Differentially abundant bacterial genera in DIO mice following RGEPH or RGDFH treatment. (**A**) Bar plot showing log_2_ fold changes in genus-level taxa between the DW group (obese mice administered distilled water) and the high-dose RGEP group (RGEPH) at week 8, as identified by Analysis of Compositions of Microbiomes with Bias Correction (ANCOM-BC). (**B**) Bar plot illustrating log_2_ fold changes in genus-level taxa between the DW group and the high-dose RGDF group (RGDFH) at week 8, also based on ANCOM-BC analysis. A log2 fold-change threshold ≥ 1, *p* < 0.05, and *q* < 0.05 were used to define statistical significance.

In the RGEP-treated mice, several key beneficial taxa were significantly enriched in a dose-specific manner: *Parasutterella* and *Akkermansia* were significantly increased in the high-dose group (RGEPH), while *Roseburia* was enriched in the medium-dose group (RGEPM) (Figs. 3A, S4A and S4B). *Akkermansia* is a strain closely associated with anti-inflammatory action and improvement of intestinal barrier function, and is attracting attention as a next-generation probiotic due to its role in maintaining mucosal integrity, improving insulin sensitivity, and contributing to overall metabolic health [32-34]. The increase of *Akkermansia* by treating RGEP observed in this study is consistent with the phenotype-based results of a previous study showing that RGEP was effective in restoring impaired immune function and intestinal barrier in a diabetic intestinal tract (DIO) mouse model, thereby maintaining intestinal immune and functional homeostasis [16]. RGEP has a targeted modulatory effect on host-protective microbial populations. Similarly, RGDF supplementation led to the enrichment of well-known SCFA-producing bacteria, including *Lachnospiraceae_UCG-004*, *Christensenellaceae* R-7 group, and *Monoglobus* (Figs. 3B, S4C and S4D). These genera are functionally significant in producing butyrate and other SCFAs, which act as primary energy substrates for colonic epithelial cells and play essential roles in suppressing intestinal inflammation and maintaining gut homeostasis [35-37] Together, these findings demonstrate that RGEP and RGDF not only enhance the abundance of beneficial bacterial taxa but also selectively promote functionally important microbial populations that are associated with anti-inflammatory activity, cholesterol metabolism, and intestinal barrier protection. This highlights their potential as microbiota-directed prebiotic interventions capable of counteracting obesity-associated dysbiosis and restoring microbial and metabolic balance in the host.

### Changes in Gut Microbial Diversity Following RGEP and RGDF Supplementation

**Fig. 4:**
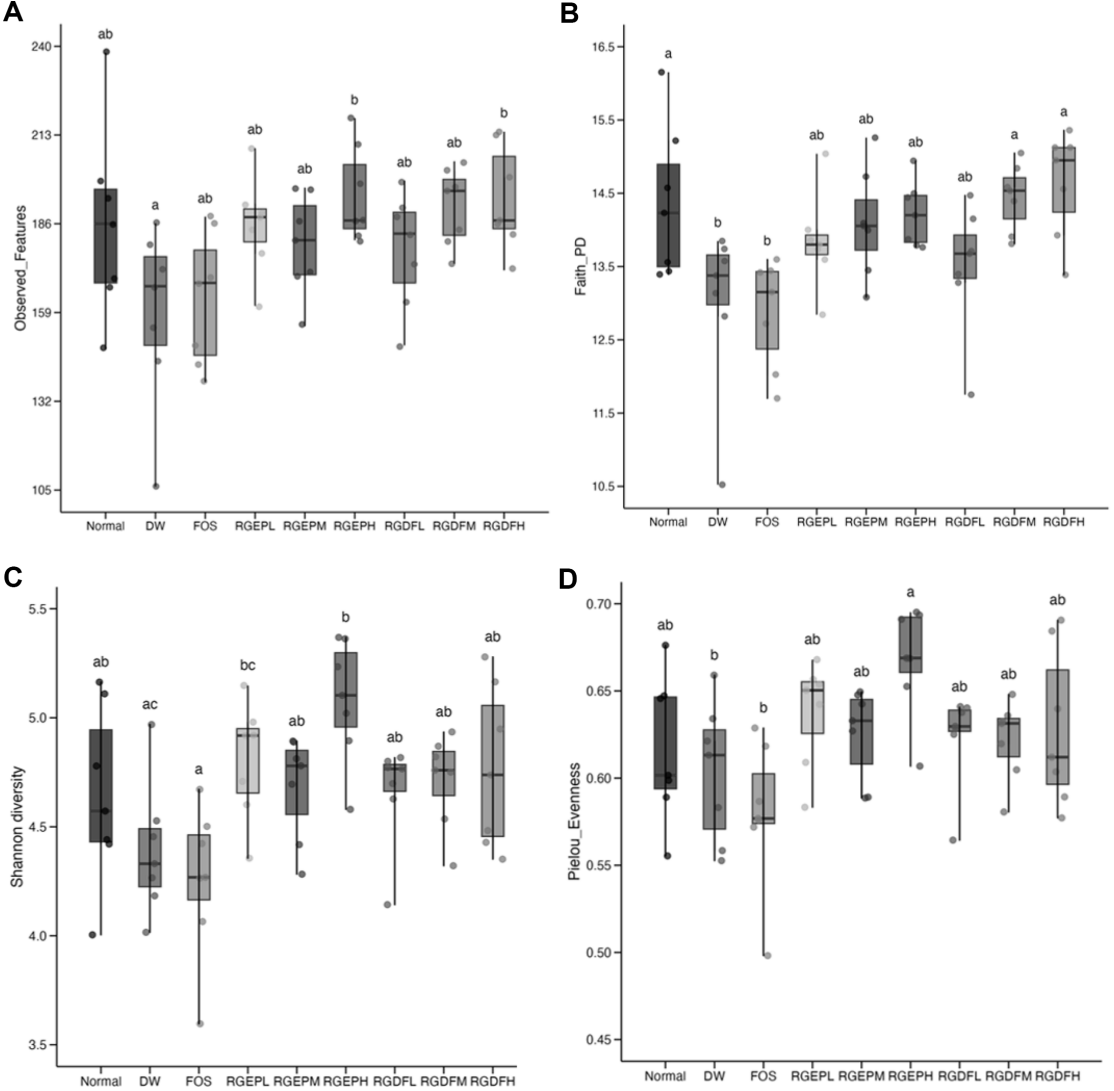
Microbial diversity metrics across experimental groups at week 8. (**A**) Observed features indicating species richness, (**B**) Faith’s phylogenetic diversity (Faith_PD) incorporating evolutionary relationships, (**C**) Shannon diversity index reflecting both richness and evenness, and (**D**) Evenness representing the uniformity of species distribution. All metrics were compared across the nine experimental groups at week 8. Statistical significance was determined using one-way ANOVA.

To quantitatively evaluate the effects of RGEP and RGDF on gut microbial diversity, we analyzed alpha diversity using four metrics: Observed Features [38], Faith’s Phylogenetic Diversity (Faith_PD) [39], Shannon Index [40], and Pielou’s Evenness [41] (Figs. 4A–4D and S5A–S5D).

At week 4, Observed features and Faith_PD (Fig. S5A and S5B) revealed that microbial richness was significantly higher in several treatment groups—namely FOS, RGEPL, RGEPH, RGDFL, and RGDFH—compared to the HFD (DW) group (*p* < 0.05), whereas no significant difference was observed between the DW and normal control groups. These findings suggest that RGEP and RGDF supplementation mitigated HFD-induced reductions in richness. In contrast, Pielou’s Evenness did not differ significantly across groups (Fig. S5D), whereas the Shannon Index showed a notable increase only in the RGDFH group (Fig. S5C), implying that high-dose RGDF may help reduce microbial dominance and promote a more balanced community. By week 8, the DW group displayed a natural rebound in richness, narrowing the gap with other groups. Nevertheless, the RGDFH group maintained significantly higher levels in Observed Features and Faith_PD (Fig. 4A and 4B), indicating a sustained enhancement in phylogenetic diversity. Interestingly, the FOS group showed a declining trend in richness over time, suggesting a diminishing long-term effect on microbial diversity. In terms of evenness at week 8, Shannon Index and Pielou’s Evenness increased significantly in most groups, except RGDFL and RGDFH (Fig. 4C and 4D), indicating that low-to-medium doses of red ginseng may be more effective in restoring microbial balance. Together, these results demonstrate that RGEP and RGDF supplementation support the recovery of gut microbial diversity under HFD conditions, with RGDFH showing the most pronounced effect on richness, whereas lower doses were more effective in improving evenness.

**Fig. 5:**
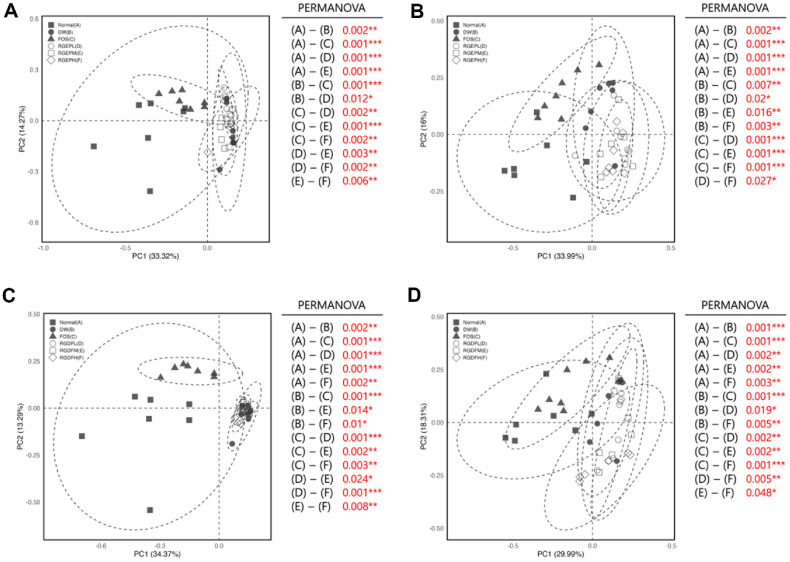
Principal Coordinate Analysis (PCoA) plot of gut microbiota based on Bray-Curtis distances, comparing different diet-fed groups. Bray-Curtis distance-based PCoA plot comparing gut microbiota composition between RGEP-fed groups with samples from (**A**) week 4 and (**B**) week 8, and between RGDF-fed groups with samples from (**C**) week 4 and (**D**) week 8. Each group is represented as follows: Normal group: ■ (filled square), DW (distilled water control group): ● (filled circle), FOS (fructooligosaccharide-fed group): ▲ (filled triangle), RGEPL (low-dose RGEP) or RGDFL (lowdose RGDF) group: □ (open square), RGEPM (medium-dose RGEP) or RGDFM (medium-dose RGDF) group: ○ (open circle), RGEPH (high-dose RGEP) or RGDFH (high-dose RGDF) group: △ (open triangle)

To assess differences in microbial community composition between samples, we analyzed beta diversity using four metrics: Bray-Curtis dissimilarity, Jaccard distance, Weighted UniFrac, and Unweighted UniFrac, visualized via Principal Coordinates Analysis. Bray-Curtis analysis, which accounts for relative abundance, revealed clear separations between the HFD control and RGEP-treated groups at both weeks 4 and 8, with stronger divergence observed in RGEPH (Fig. 5A and 5B) [42]. The RGDF groups showed a similar pattern, with RGDFH at week 8 exhibiting the most distinct shift from the obesity model (Fig. 5C and 5D). The Jaccard index, which focuses on species presence or absence [43], detected significant group differences for both RGEP and RGDF at both time points (*p* < 0.0, with greater divergence at week 8, suggesting progressive changes in microbial composition over time with red ginseng supplementation (Fig. S6). The Weighted UniFrac metric [44], which incorporates both phylogenetic relatedness and abundance, showed that RGEPH and RGDFH groups deviated most strongly from the obesity model at both weeks 4 and 8 (Fig. S7). In contrast, Unweighted UniFrac [45], which considers phylogeny without abundance, also revealed significant differences between RGEP/RGDF and HFD groups (*p* < 0.05), with effects becoming more pronounced at week 8. In the RGEP-treated groups, PCoA plots demonstrated clear separation from the normal and FOS groups at both week 4 (Fig. S8A) and week 8 (Fig. S8B), indicating that RGEP altered the phylogenetic composition of the gut microbiota. However, no substantial divergence was observed among the RGEP dose groups (RGEPL, RGEPM, RGEPH), suggesting a dose-independent response. For RGDF-treated groups, a similar pattern was observed at week 4 (Fig. S8C), where all RGDF doses clustered away from the normal and FOS groups, but without clear differentiation among the RGDFL, RGDFM, and RGDFH groups. Notably, at week 8 (Fig. S8D), the RGDFH group (△) exhibited a distinct shift in clustering compared to both control groups and lower-dose RGDF groups, indicating a dose- and time-dependent effect specific to high-concentration RGDF. Interestingly, the DW group (●) remained in proximity to the RGEP and RGDF clusters across all time points, showing limited separation in Unweighted UniFrac space, a finding that contrasts with trends observed in other β-diversity metrics and warrants further investigation. Together, these findings suggest that RGEP and RGDF supplementation leads to a significant, dose-dependent restructuring of the gut microbiota. They influence not only the richness and evenness of microbial communities but also drive compositional and phylogenetic shifts. The pronounced effects observed with higher doses underscore the importance of dosage and treatment duration in modulating gut microbial ecosystems.

### Functional Modulation of Gut Microbial Metabolic Pathways by RGEP and RGDF in DIO Mice

To investigate the functional impact of red ginseng supplementation on gut microbial metabolism, we performed PICRUSt2-based metagenomic pathway prediction using 16S rRNA gene sequencing data. A heatmap of the top 20 differentially abundant pathways (Z-score normalized) at week 8 revealed distinct clustering patterns across treatment groups [46].

In the RGEP-treated groups, several microbial metabolic pathways predicted to be associated with SCFA production showed increased relative abundance. Notably, this included PWY-7211 (propionate biosynthesis), FUCCAT-PWY (fucose degradation), and P163-PWY (ethanol oxidation) (Fig. S9A). Among them, FUCCAT-PWY—responsible for fucose degradation—was more prominent in RGEP groups, regardless of concentration. Although this pathway is microbiota-derived, its substrate L-fucose has been previously linked to metabolic improvement via AMPK activation, indicating a potential role in lipid regulation [47]. In addition, pathways such as PWY-6892, PWY-7371, and P562-PWY, implicated in host–microbiome interactions and immune-related functions, were also relatively enriched in RGEP groups. These findings align with the known anti-inflammatory and immunomodulatory effects of red ginseng [48-50].

Similarly, the RGDF-treated groups showed elevated relative abundance in pathways such as P124-PWY and LACTOSECAT-PWY, which are predicted to contribute to SCFA production and support gut barrier integrity and inflammation control [51, 52] (Fig. S9B). In the RGDF-treated groups, functional enrichment was observed in SCFA- and carbohydrate-associated pathways, such as P124-PWY, LACTOSECAT-PWY, and GLUCARDEG-PWY (Fig. S10). FUC-RHAMCAT-PWY was consistently enriched across both RGEP and RGDF groups, suggesting a shared microbial mechanism involving fucose and rhamnose metabolism. Notably, several pathways related to host–microbiome signaling and immune regulation, including TEICHOICACID-PWY and PWY-6892, also showed elevated activity in the RGDF groups, albeit to a lesser extent than in the RGEP groups (Fig. S10). These results suggest that RGDF, like RGEP, may contribute to the restoration of metabolic balance by modulating both microbial metabolism and immunoregulatory pathways.

## Discussion

The present study investigated the effects of RGEP and RGDF supplementation on gut microbiota composition, microbial diversity, and metabolic functions in DIO mice. Our findings demonstrate that RGEP and RGDF modulated gut microbiota composition by increasing beneficial bacteria, enhancing SCFA metabolism, and reducing pro-inflammatory bacterial taxa. These effects suggest a potential role for red ginseng components in gut microbiome regulation and metabolic health improvement.

By integrating taxonomic, diversity, and functional analyses, our study provides a comprehensive characterization of microbiota-level changes. The use of alpha and beta diversity indices allowed robust assessment of microbial community shifts. PICRUSt2-based predictions adds insights by revealing potential metabolic improvements, linking microbiota shifts to physiological outcomes. The study compares RGEP and RGDF, demonstrating their differential effects on gut microbiota, particularly RGDF’s strong influence on microbial evenness and SCFA metabolism.

However, it should be noted that functional inferences such as enhanced SCFA metabolism were derived from predictive metagenomics, not direct metabolite quantification. As such, conclusions regarding microbial metabolite production remain speculative and should be validated by targeted metabolic profiling in future studies.

This analysis focused solely on microbiota-level responses derived from a larger collaborative study. Host physiological parameters, including body weight, adiposity, serum lipid levels, and fecal SCFA concentrations, were assessed using the same animal cohort and reported elsewhere [16]. Future integrative analyses that connect microbial shifts to systemic metabolic phenotypes will be essential for clarifying the physiological relevance of the observed microbial changes.

Interestingly, microbial responses to RGEP and RGDF were not always dose-dependent, particularly in alpha diversity metrics (Fig. 4A–4D). This non-linearity may reflect threshold effects or microbial competition, underscoring the complexity of ecosystem-level interactions. Furthermore, RGEPH and RGEPL groups showed closer resemblance to each other than to RGDF-treated groups in specific bacterial profiles (Fig. 3A), indicating that microbial shifts are influenced not only by dosage but also by compound-specific characteristics. These patterns may stem from differences in ginsenoside and fiber content between RGEP and RGDF, highlighting the need for further compositional and metabolomic analyses.

While FOS is widely reported to promote beneficial bacterial growth, the effects observed in our study were less pronounced than expected [27-29]. Several factors may contribute to this outcome, including the relatively low dose of FOS used, the limited duration of intervention (8 weeks), and possible strain-specific responses in the gut microbiota of the C57BL/6 mice used. Previous research has shown that the efficacy of FOS varies by host genotype and baseline microbiota composition, which may have limited its impact in our model [53].

In conclusion, RGEP and RGDF supplementation modulated gut microbiota composition, increased microbial diversity, and altered predicted metabolic pathways in DIO mice. Although these microbiota-level changes are promising, further research is needed to assess their translation to host metabolic outcomes. Red ginseng components may serve as candidate prebiotic interventions for restoring gut homeostasis and supporting metabolic health, but their anti-obesity potential remains to be confirmed through integrated multi-omic and translational studies.
